# Genome-Wide Association Study Reveals Single Nucleotide Polymorphisms Associated with Tail Length and Tail Kinks in Piglets

**DOI:** 10.3390/vetsci12030198

**Published:** 2025-02-24

**Authors:** Katharina Gerhards, Christiane Egerer, Sabrina Becker, Hermann Willems, Petra Engel, Sven Koenig, Gerald Reiner

**Affiliations:** 1 Clinic for Swine, Justus Liebig University Giessen, Frankfurter Strasse 112, 35392 Giessen, Germany; katharina.gerhards@vetmed.uni-giessen.de (K.G.); christiane.egerer@vetmed.uni-giessen.de (C.E.); sabrina.becker-2@vetmed.uni-giessen.de (S.B.); hermann.willems@vetmed.uni-giessen.de (H.W.); 2 Institute of Animal Breeding and Genetics, Justus-Liebig-University of Giessen, 35390 Giessen, Germany; petra.engel@agrar.uni-giessen.de (P.E.); sven.koenig@agrar.uni-giessen.de (S.K.)

**Keywords:** tail biting, GWAS, animal welfare, swine

## Abstract

Tail docking is still used in pigs to reduce the prevalence of tail biting, although it is purely symptomatic and contrary to animal welfare. Genetic selection for shorter tails might, however, help to avoid tail docking and has therefore been proposed. A genetic basis for tail length is known for many species. Variability in tail length, including moderate heritability, has also been demonstrated in pigs. The aim of the present study was to identify genetic markers for tail length and to define candidate genes. To this end, 140 piglets were phenotyped and genotyped at 3 days of age and a genome-wide association study was performed. Seven SNPs were mapped on chromosomes 1, 2, 6, 11, and 15. Two linked SNPs on chromosome 2 resulted in a functional amino acid exchange. The genotypes at the SNPs were only associated with small differences in relative tail length of up to 16.5% (short genotype versus long genotype at SSC15), but at the same time with the occurrence of malformations in the form of tail kinks. The small effect size and the association between tail length and tail kinks, together with the generally pure symptomatic effect on tail biting, argue against the applicability of selection for shorter tails in pigs.

## Simple Summary

Tail docking is a symptomatic yet effective and widely used, but animal unfriendly, method of reducing the prevalence of tail biting. This led to the idea of genetically shortening tail length through selection. However, this procedure can lead to side effects in the form of tail kinks. The aim of the present study was a genome-wide search for gene markers and candidate genes associated with tail length. The identified marker alleles differed only slightly in their associated tail length. At the same time, the close genetic association between shorter tails and malformations was confirmed. The results of the present study argue against the benefits of genetic selection for shorter tails in pigs.

## 1. Introduction

Although tail docking in piglets is only a symptomatic measure to reduce the incidence of tail bites [1,2] and routine tail docking has been banned by EU legislation since 2008 [2], the practice is still widespread. The risk of tail biting is reduced with shorter tails [2,3,4,5], but it is still unclear whether this is due to increased sensitivity of the stump and consequent tail withdrawal [6,7,8,9] or simply poorer accessibility [4,7,8]. The procedure is painful and causes distress and harm to the animals [9,10,11,12]. Several research groups are now promoting tail shortening by genetic selection to at least reduce the incentive and accessibility to bite [13,14], thus reducing one factor in the multifactorial occurrence of tail biting. However, several studies have raised concerns that genetic tail shortening in pigs [13] and other species [15,16,17,18,19,20] is associated with the occurrence of deformities such as kinks. For example, in this study, up to 28% of piglets with the shortest tails had kinks of varying severity.

Both tail length and the occurrence of kinks are significantly associated with sow and boar [13], and a heritability of 0.42 was estimated for tail length [14]. Based on this evidence for a genetic component of tail length and kinks, the aim of the present study was to reveal underlying effects in the genome by analysing whole genome sequence (WGS) data using a genome-wide association study (GWAS). Little information is available on candidate genes related to tail length and occurrence of tail malformations in pigs, but a large number of candidate genes are known, some of which may be causally involved in different animal species; therefore, we paid special attention to such candidate genes.

## 2. Materials and Methods

### 2.1. Animals and Clinical Examination

In the following study, 348 animals from an existing resource population from previous studies were used [14].

The piglets were all from the commercial piglet production of the ‘Oberer Hardthof’ Teaching and Research Station of the Department of Agricultural Sciences at the Justus Liebig University Giessen (OH).

Research on tail length by the Institute of Animal Breeding and Genetics at Justus Liebig University Giessen on the piglets of OH over the past few years has already shown that the herd is segregating in terms of tail length [14].

The piglets came from 24 litters of 6 Pietrain boars with 21 sows from four internal lines crossed with Topigs Norsvin (TN70). Within 14 rounds, the boars were mated with 1 to 7 sows. Three sows had two litters with two different boars. Litter size was 17.54 ± 3.7 piglets in total and 16.7 ± 3.27 piglets born alive.

On the 3rd day of life, all piglets were weighed, measured, and photographed by the same person. A tape measure was used to measure tail length and body length in cm. Body length was measured from the base of the ear to the tip of the tail. The absolute length of the tail was measured from the tip of the tail to its base at the body, following the whole tail in its median line. Relative tail length (tail length/body length × 100) was calculated and used for further analysis, as the difference in overall size and weight of piglets has a major influence on the parameter absolute tail length.

In addition, it was recorded whether a kinked tail (number, degree of kink (30°, 60°, 90°, 180°)) or other anomalies were present.

Both the suckling piglets and the weaners were examined several times during the trial period by a trained observer for signs of tail biting (bleeding, tissue damage, bite marks with counter bite) and were subject to daily animal checks by the farm personal. The animals were also given the usual zootechnical treatment and vaccinations at this farm: day 1: teeth clipping, ear tacking; day 2: iron application (Ursoferran, 200 mg/mL, Serumwerke Bernburg, Bernburg, Germany); day 6: castration; and day 25: vaccination against PCV2 and Mycoplasma hyopneumoniae (Porcilis PCV Mhyo, MSD, Munich, Germany).

Due to the high prevalence of tail lesions on the farm, all piglets were docked directly after the examination on day 3. The tail was first measured and the last third was removed by cauterisation, regardless of the presence and position of kinks.

All experiments were approved by the Animal Welfare Officer of the Ethics Committee of the Justus Liebig University, Giessen, Germany, with the reference JLU_kTV_4_2021. The work was carried out in accordance with the animal welfare guidelines of the Justus-Liebig University and no ethical approval was required according to the German Animal Welfare Act. Relevant guidelines and regulations were followed for all methods.

Of the 348 piglets available, 140 were selected to ensure that the extremes of relative tail length were evenly distributed and that all kinks were included.

### 2.2. DNA Extraction and Sequencing

Docked piglet tails were used as sample material for DNA extraction with the smart DNA prep (m) kit (Analytik Jena, Jena, Germany).

DNA was quantified using the Qubit dsDNA broad range assay kit (Invitrogen, Thermo Fisher Scientific, Waltham, MA, USA) on the Qubit Flex Fluorometer (Invitrogen, Thermo Fisher Scientific, Waltham, MA, USA) and diluted to a uniform concentration of 50 ng/μL.

Whole genome sequencing was performed on an Illumina NovaSeq 6000 (Illumina, San Diego, CA, USA) generating 150 bp paired-end reads of 15× coverage.

Further sequencing data preparation included the demultiplexing of all libraries for each sequencing lane using Illumina bcl2fastq v2.20 software (https://emea.support.illumina.com/sequencing/sequencing_software/bcl2fastq-conversion-software.html; accessed on 21 February 2025) and the clipping of sequencing adapter remainders from all reads. Reads with a final length of <20 bases were discarded.

The mean read depth per sample was 16.01, with a maximum and minimum read depth per sample of 23.00 and 11.23, respectively.

### 2.3. Bioinformatics Workflow

The .bz2 compressed fastq files received from the sequencing company were decompressed and converted to .gz compressed fastq files for further analysis.

#### 2.3.1. OVarFlow Pipeline

The available adapter-clipped raw data were passed to the open-source workflow OVarFlow for further bioinformatic analysis. This workflow is used for the variant discovery of single nucleotide polymorphisms (SNPs) and insertion/deletion polymorphisms (indels) in model and non-model organisms [21]. The workflow allows the automation, documentation, and reproducibility of the individual evaluation steps.

The reference genome and annotation Sscrofa11.1 (GCF_000003025.6) were used for the implemented alignment, variant calling, and annotation. The min sequence length was set to 1.

Due to the given hardware resources in combination with the existing data structure, a modification of the heap size and bwa memory was made which deviated from the standard settings (initial standard settings are shown in brackets in Supplementary Table S1).

The elements of the remaining analysis steps and the programmes and versions used in the OVarFlow workflow correspond to those already described in [22].

#### 2.3.2. Genome-Wide Association Study (GWAS)

The preparation of the data output from OVarFlow for the GWAS and the actual execution of the GWAS was carried out in R, version 4.2.1 [23]. RStudio (version 2024.12.1-563) [24] was used as the graphical user interface.

The annotated VCF file output at the end of the OVarFlow workflow was converted to binary PLINK format using the PLINK package, version 1.9 [25]. An initial quality control of the genotype data was performed using PLINK. Only variants and individuals meeting the following filtering criteria were kept for GWAS:

- Missingness per marker < 0.01;
- Missingness per individual < 0.1;
- Minor allele frequency (MAF) > 0.05;
- Hardy–Weinberg equilibrium (HWE) *p* > 0.000001.

At the gonosomal level, the GWAS analysis was extended to the X chromosome. The pseudoautosomal region (PAR, SSC21) and the X-specific region (X, SSC19) on the X chromosome were analysed separately. The PAR is the region where recombination between the X and Y chromosomes occurs and is associated with early embryonic development [26,27].

The pseudoautosomal boundary (PAB) was defined as described in [28] and transferred to the used reference genome Sscrofa11.1 by sequence comparison using NCBI blastn [29,30]. Genome data were extracted using the --split-x command in PLINK. Quality control was performed using the same method as used for the autosomes.

A total of 14,159,238 variants were detected on the autosomes and the X-specific and PAR regions of 140 sequenced pigs.

A reduced SNP dataset was generated to check for any population structure that might be present. SNP pruning was based on linkage disequilibrium and pairwise genotypic correlation using PLINK.

Principal component analysis (PCA) was performed on the reduced dataset using TASSEL, version 5.2 [31]. The graphical visualisation was carried out using the R package ggplot2, version 3.5.1 [32] (Supplementary Figure S1). PCA is used primarily for dimensionality reduction, and many individual components would have to be considered to explain over 90% of the variance of the target characteristics. The first principal components together explained only 12.3% of the total variance. Therefore, to account for the stratification of the data, the distances from the kinship analysis were used in the GWAS model.

TASSEL was also used to create a kinship matrix to account for kinship relationships in further GWAS analysis.

The actual GWAS was conducted by GAPIT, version 3 [33], using the BLINK model [34]. For this purpose, genotype data were converted from PLINK to the hapmap format using TASSEL. Litter size, litter number (parity of the sow), and sex were used as fixed effects.

By using relative rather than absolute tail length, stronger correlations between factors that may influence growth and body weight, and thus absolute length [13], were excluded a priori. Nevertheless, litter size, parity, and sex were included to account for possible residual effects [13].

Bonferroni-corrected genome-wide and chromosome-wide significance thresholds (Psig: 3.53 × 10^−9^) were set for a significance level of α = 0.05. In addition, a less stringent correction was applied and defined as the suggested threshold (Psugg: 1 × 10^−5^).

Manhattan plots and quantile–quantile plots were generated using the R package qqman, version 0.1.8 [35].

#### 2.3.3. Variant Effect Prediction

Functional annotation of previously found genetic variants was performed using snpEff, version 5.0 [36], and Ensembl Variant Effect Predictor (VEP), release 113 [37]. Information is provided on the genetic coordinates, effects, and impacts of each variant.

For non-synonymous mutations, the SIFT (sorting intolerant from tolerant) score was determined, which predicts whether an amino acid substitution could affect protein function based on sequence homology and the physical properties of the amino acids [38].

### 2.4. Statistical Analysis of SNP Effects on Tail Traits

The effects of the genotypes of the significant SNPs from the GWAS on tail characteristics were tested using ANOVA (metric data) and a general linear model (binary data) in IBM-SPSS, version 27 (Statistical package for Social Sciences, IBM, Munich, Germany). SNPs were only included if the negative decadic logarithm of significance exceeded 8.45. We tested the effects of the SNPs not only on the trait significant in the GWAS but also on all relevant parameters: relative tail length, presence of kinks (0/1), and kink grades. The rationale for this approach was the assumption that, because of the correlation between tail length and kinks, the involved gene loci might affect different tail characteristics simultaneously.

### 2.5. Annotation of Potential Candidate Genes

Positional candidate genes were searched for using the NCBI Genome Data Viewer with the reference genome Sscrofa11.1 (https://www.ncbi.nlm.nih.gov/gdv/browser/genome/?id=GCF_000003025.6; accessed on 21 February 2025).

A gene was considered to be a candidate gene if at least one significantly associated variant was located in the gene or if the gene was located within a window of 1 Mbp upstream and downstream of the respective SNP.

The physiological functions of the potential candidate genes were inferred using information from the NCBI database [39], GeneCards [40], PathCards [41], KEGG [42], MGI [43], and DAVID [44].

## 3. Results

### 3.1. Phenotypes

The 140 piglets for the GWAS were selected from the entire cohort of 348 piglets that were available on the farm. The aim was to increase variation while reducing the number of animals for genome-wide sequencing. The phenotypes of all piglets and the piglets selected for GWAS are shown in Table 1. The main effect factors, such as sex, parity, and litter size, showed no differences between the two groups. As the selection of piglets for the GWAS was based on extremes, the piglets with the shortest and longest tails in the whole cohort were fully included in the GWAS group. As a result, the variance of the values increased despite the smaller number of animals (see standard deviation). The absolute lengths are also shown in Table 1, as are the relative tail lengths. Only relative tail length was used in the GWAS to minimise confounding by piglet weight and size. All further information refers only to 140 piglets from the GWAS. As all 348 animals with kinked tails were selected, the relative number of piglets with kinked tails and the degree of kink increased in the GWAS group compared to the total group (Table 1).

The absolute tail length in the GWAS group was 9.91 ± 2.11 cm (mean ± SD), with a minimum of 6.26 cm and a maximum of 12.0 cm. The relative tail length varied between 20.8% and 31.0% of the total piglet length, with a mean of 26.7% (SD = 2.13). In total, about 30% of the piglets had kinked tails. In the group of the 20% piglets with the longest tails, 7% had kinked tails. In the group of 20% piglets with the shortest tails, 54% had kinked tails (Table 2).

### 3.2. GWAS

The GWAS identified seven significant SNPs, of which four were genome-wide significant after Bonferroni correction (*p* ≤ 3.53 × 10^−9^; negative log(*p*) ≥ 8.45). A further 3 SNPs out of 376 suggestive SNPs (*p* ≤ 1 × 10^−5^; negative log(*p*) ≥ 5.00) were classified as functional and thus included in further analysis. The SNPs were associated with three different phenotypes (relative tail length, kinks01, kink grades). All SNPs were already known and available under their rs ID (Table 3).

**Figure 1:**
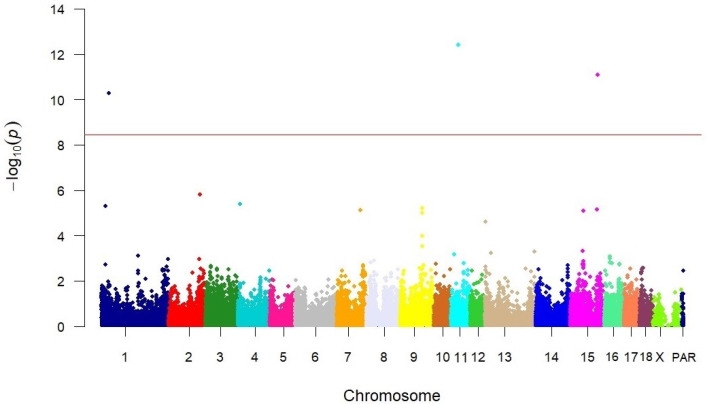
Exemplary Manhattan plot of SNPs associated with tail kink grades.

**Figure 2:**
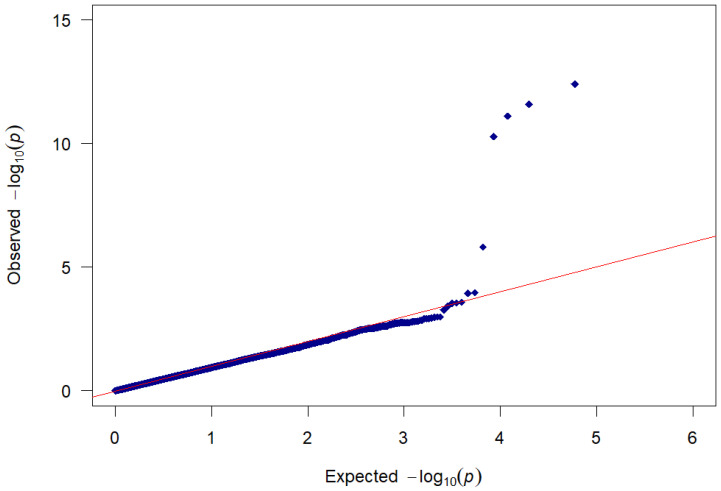
Q-Q plot corresponding to Figure 1.

The Manhattan plot summarises the effects of SNPs associated with tail kink grades (°). Genome-wide effects for this trait were found on chromosomes 1, 11, and 15. Suggestive functional effects were found on SSC2 (Figure 1). The QQ plot and genomic inflation factor (λ = 1.09) did not indicate population stratification and/or cryptic relatedness between animals (Figure 2).

The four SNPs significantly associated with relative tail length or kinks in the GWAS were located in intergenic regions and the intron of the JAK1 gene, respectively (Table 3). As modifiers, they were not expected to have a direct effect. The SNP on SSC1 was flanked by LOC106507477 and LOC102166614. Serum/glycocorticoid-regulated kinase 1 gene (SGK1) was located 933 kbp 5′ to the SNP, ribosomal protein S12 (RPS12) was located 221 kbp 3′, and the cellular communication network factor 2 gene (CNN2) was located 974 kbp 3′ to the SNP.

The three SNPs on SSC2 were completely linked. Two of them led to an amino acid exchange in the protocadherin A1 (PCDHA1) gene, the third SNP was a variant in the 5′ UTR with limited effect (Table 3). An amino acid exchange from Serine (Ser) to Arginine (Arg) was interpreted by SIFT as deleterious, the other, from Lysine (Lys) to Arg, was tolerated. Ser is a small and polar, but in terms of its acidity neutral amino acid, Lys is a polar and basic amino acid. Both were found to be exchanged to Arg, a polar and highly basic amino acid.

The SNP on SSC6 was located within intron 1 of the Janus kinase 1 (JAK1) gene (Table 3). The SNP on SSC11 was located in an introgenic region flanked by LOC100515564 5′ and protocadherin 17 (PCDH17), 7.8 Mbp to the 3′ end. The SNP on SSC 15 was flanked by the pleckstrin homology domain containing M3 gene (PLEKHM3), 30 kbp 5′, and LOC100154892 3′ to the SNP. The frizzled class receptor 5 gene (FZD5) was located 285 kbp 3′ to the SNP.

### 3.3. Effects of the SNPs

The seven SNPs associated with tail characteristics were located on chromosomes 1, 2 (n = 3), 6, 11, and 15. All three possible genotypes were present at all SNPs, except for the SNP on SSC6, where only two genotypes were found. The SNPs on SSC1, 2, and 15 were significantly associated with relative tail length and kink grades in the ANOVA and with the occurrence of kinking (0/1) in the general linear model. The genotypes with the shortest tails at the individual SNPs were only 0.06% = 0.2 mm (SSC1), 2.17% = 7.8 mm (SSC2), 0.26% = 0.9 mm (SSC11), and 4.26% = 15.2 mm shorter than the average tail (26.7% = 95.8 mm). For the SNP on SSC6, the tails of the shortest homozygous genotype were 0.42% = 1.5 mm shorter than the average tail.

However, the genotypes with the shortest tails had 35.8% (SSC1), 65% (SSC2), 79% (SSC11), and 76% (SSC15) more piglets with kinked tails than the genotypes with the longest tails. At the same time, the degree of kinking in the genotypes with the shortest tails was increased by 17.6° (SSC1), 46° (SSC2), 129° (SSC11), and 183° (SSC15), respectively.

The association of the SNP on SSC11 with relative tail length was at the significance threshold, while its association with kinks was highly significant. The two available genotypes of the SNP on SSC6 were associated with relative tail length but not with kinks. The genotypes associated with the shortest tails were always associated with the highest proportions of kinks and the highest kink grades. With the exception of the SNP on SSC1, the homozygous genotypes with the strongest association with kinks and relative tail length were always significantly less frequent than the homozygous genotypes associated with longer tails with less kink exposure and less frequent than the heterozygous genotypes. With the exception of the SNP on SSC1, kinks were never observed in the most frequent genotype (Table 4).

Each of the SNPs on the different chromosomes had one genotype that was associated with the shortest tails, but also the highest probability of kinks. When comparing the genotypes of the SNPs on SSC1, 2, 11, and 15, most piglets had none of the four different genotypes associated with short tails and kinks (Table 5). Some piglets had these genotypes from one SNP, some from two, and others from three of the four SNPs. No piglet had the short tail/kink associated genotypes of all four SNPs. As the number of short tail/kink-associated genotypes increased, the tail length decreased significantly by 1.9% = 6.8 mm and the incidence and degree of kinking increased significantly by 72% and 126°, respectively (Table 5).

**Figure 3:**
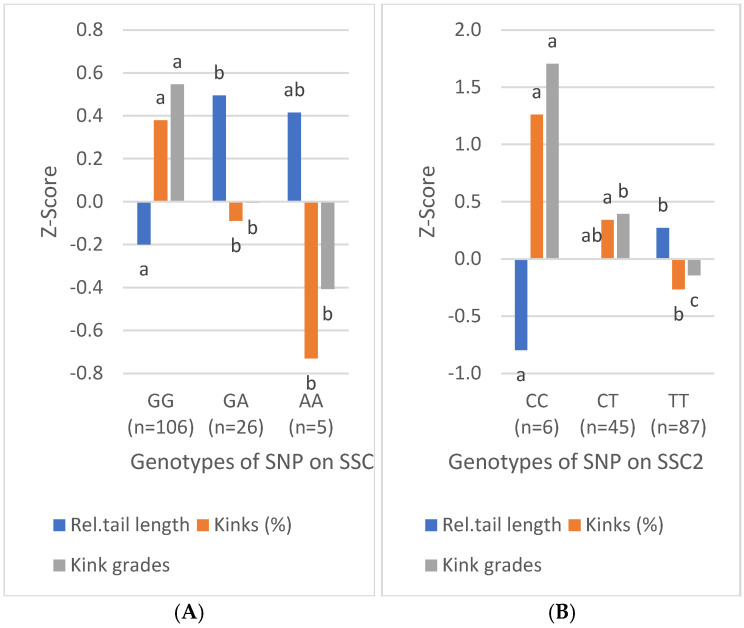

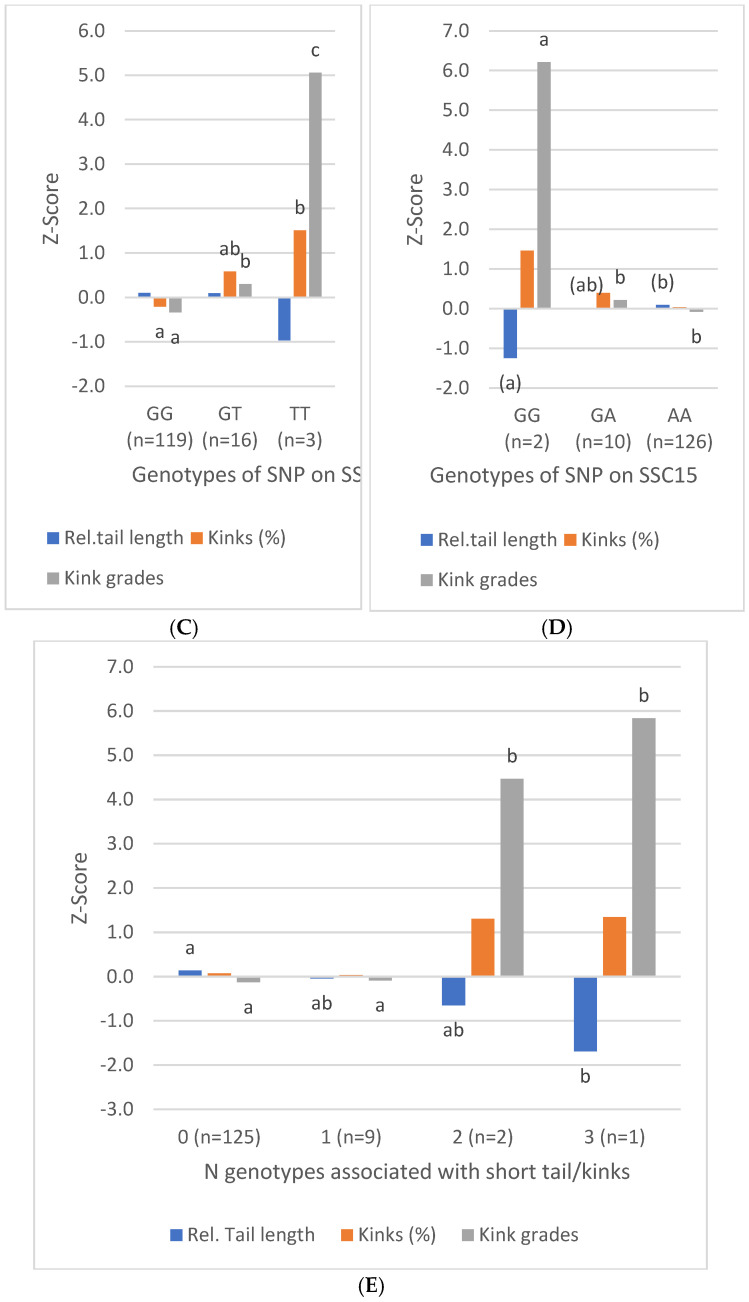
Effects of SNP genotypes on relative tail length, prevalence, and severity of tail kinks in piglets. (**A**) SNP on SSC1; (**B**) SNP on SSC2; (**C**) SNP on SSC11; (**D**) SNP on SSC15; (**E**) number of genotypes associated with short tails and kinks. Values with different letters are statistically significant (*p* < 0.05).

The effects of individual SNPs and the combination of SNP genotypes on relative tail length and kinks are particularly evident after Z-transforming the values (Figure 3). With the exception of the SNP on SSC1, the rare genotype is always associated with shorter tails, higher kink prevalence, and higher kink degrees. The strongest effects are shown by the extreme genotypes of the SNPs on SSC11 and SSC15. With the exception of the SNPs on SSC1 and SSC2, the associations with kink were significantly stronger than those with tail length.

## 4. Discussion

The tail docking of piglets within the first days of life is still practiced in many countries to reduce the prevalence of tail biting [2,5]. Although it is not fully understood why, shorter tails do significantly reduce the risk of biting [1,2]. However, this is largely a symptomatic approach as it does not eliminate the underlying multifactorial causes that lead animals to bite [45] but simply makes access to the tail more difficult. In addition, tail docking causes pain, suffering, and harm to the animals treated [10,11]. The effect of tail docking on growth and performance has been evaluated inconsistently by different authors. On the one hand, the duration and magnitude of the stress response during tail docking do not seem to be sufficient to affect growth and performance [46,47]. Other authors found no or only small effects [48]. Nevertheless, the behaviour of the treated piglets is indicative of pain. Signs of pain, discomfort, and anxiety are visible during the acute phase but also days and weeks after tail docking [49]. These include increased and prolonged vocalisation at higher frequencies; increased escape behaviour; changes in posture, including prolonged periods of standing with head down; sitting and tail wagging; squatting, crouching, and cowering; and an increased stress response (cortisol) compared to sham-treated piglets [49,50,51]. In addition, the wound can be an entry point for infections [10].

In principle, tail length can be reduced by genetic selection to avoid tail docking, in addition to husbandry and management measures to reduce the need for tail docking [13,14]. To date, there is only a limited database from a few studies in which the absolute tail length of pigs in different age groups has been investigated [8,13,14,52]. Preliminary tests showed that the relative tail length of the 348 phenotypically examined piglets varied between 20.8 and 31% of the total body length (tail tip to ear base) depending on the sow and boar. The heritability for tail length was estimated to be 0.42 [14], suggesting a strong genetic basis for the trait. In the present study, seven SNPs were identified by GWAS that were associated with relative tail length at different levels of significance. The SNPs were located on chromosomes 1, 2, 6, 11, and 15. The largest difference between the minima and maxima of the homozygous genotypes in terms of absolute tail length was 39 mm/16.7% (SSC15). However, the average difference was only 4.3% = 15.3 mm. Even the combination of the four SNPs with all three genotypes did not lead to a greater reduction in tail length. It is therefore questionable whether these relatively small differences could actually have a significant effect on biting behaviour. Thodberg et al. [4] showed a largely linear relationship between tail length after docking and the prevalence of tail biting. A reduction in tail length (by docking) of 24% (maximum 16.7% in the present study) was associated with a small reduction in biting, but due to the small number of animals, this effect only became significant at a reduction of 61%. It also remains unclear what role the actual length and the possibly higher sensitivity of the stump play in this effect.

While the effects on tail length were relatively small, there was a strong association between tail length reduction and the occurrence of kinks. Phenotypically, 41 out of 348 piglets in the total cohort (12%) had kinked tails. Kinks occurred in 5% of the piglets in the highest 20th percentile (highest relative tail length) but in 28% of the animals in the lowest 20th percentile (lowest relative tail length) in the total cohort of 348 piglets. As all piglets with kinks were included in the GWAS, 41 out of 140 piglets in the GWAS (30%) had kinked tails. Therefore, the corresponding 20th percentiles of the 140 GWAS animals inevitably showed higher proportions of kinks (7% and 54%, respectively).

The severe impairment of the tails by kinks (qualitatively and quantitatively) was also evident from the SNPs in the present study. In particular, for the SNPs on SSC11 and SSC15, the homozygous genotypes differed by 129° and 183°, respectively. At the same time, 79% and 86% more animals were affected by kinks in the sensitive genotype than in the respective insensitive genotype. The prevalence of kink was always higher in the short-tailed animals.

Short-tailedness, also known as brachyury, occurs in many vertebrate species, although the genetic causes of this phenomenon are heterogeneous, with additive polygenic (e.g., [53]) as well as Mendelian mono- and oligogenic factors (e.g., [18]). In general, variation in tail length, both across generations and spontaneously, is not uncommon in vertebrates. Phylogenetic studies suggest that tail shortening or loss has evolved independently in different animal species, although the evolutionary ‘shortening mechanisms’ have not been elucidated [18]. Furthermore, neither the full mechanism nor the complexity of tail length inheritance is fully understood, especially when considering the additional evidence for additive genetic effects.

Short tails also occur in pigs [52,54,55]. However, there is a considerable lack of scientific studies dealing with the genetic variation in tail length. Preliminary studies [14] suggest moderate to high heritability and therefore a strong additive genetic basis. Apart from pigs, quantitative genetic parameter estimates for tail length are available for several non-livestock species, such as toque macaques [56] and mice [57]. The heritabilities were 0.67 and 0.46, respectively.

The T gene is the most prominent gene associated with variations in tail length but also with spinal deformities in various vertebrates, including mice, zebrafish, dogs, cats, and cattle [18,19,58,59,60,61]. The T gene encodes a transcription factor that is expressed specifically during early embryonic stages and influences mesoderm development and chorda dorsalis differentiation [60]. Herrmann et al. [16] detected a deletion of 200 kb as the causal candidate mutation for extremely short tails in mice that caused early embryonic death when homozygous. Also, in cats, mutations the T gene are responsible for short-tailed breeds such as Manx cats. Mutations in exons 8 and 9 of the feline T gene can cause short tails as well as changes in spinal structures [18]. Hytönen et al. [17] identified a mutation in exon 1 of the canine T gene that was associated with short-tailed phenotypes and embryonic mortality. In cattle, Kromik et al. [19] identified a T-gene influence on the number of cervical and caudal vertebrae, as well as vertebral shape. T-gene sequence analysis of the affected animals revealed a spontaneous synonymous point mutation (c.196A > G) within the highly conserved T-box, causing a spinal anomaly (i.e., vertebral and spinal dysplasia (VSD)). Kromik et al. [19] provided the first evidence for the direct influence of the T protein (Brachyury) on the number of cervical vertebrae. Accordingly, the T gene may also be involved in the expression of the present phenotypes of tail shortening and kinks in pigs.

In pigs (Sscrofa11.1), the T gene is located on SSC1 in the range from 2,717,165 to 2,725,561. In this region, no association with tail length or curvature was found in the present study. This is in line with the results of Klein et al. [52], who found only minor genetic differences in several analysed genome sections of the brachyury or T gene on SSC 1. However, it cannot be excluded that the relatively small number of animals in this study may have played a role. 

Shortened tails are also known in other animal species, which cannot be attributed to the T gene. In addition to the influence of the T gene, Greco et al. [62] and Wilm et al. [63] identified sex-specific QTL effects on tail length in mice on chromosomes 9 and 15. Several other genes, such as the paired box 1 gene (PAX1) and the wnt family member 3A (WNT3A), influenced tail length in mice [62,63]. PAX3 has been associated with curly tails and neural tube defects in mice [64]. In Asian cats, variations in tail length were also due to the influence of the HES family bhlh transcription factor 7 gene (HES7) [20]. A SNP and an insertion within or near the homoeobox B3 gene (HOXB13) have been associated with short tail length in Merino sheep [65]. The LIN-28 homolog A gene (LIN28A) is involved in mammalian tail length [66]. The vascular endothelial growth factor A (VEGFA) [67], ankyrin repeat domain containing 11 (ANKRD11), activin A receptor type 2B (ACVR2B), and secreted frizzled-related protein 2 (SFRP2) are candidate genes for the shortened tail in an endangered Korean dog [68]. The precise regulation of homoeobox B6 gene (HOXB6) appears to be essential for proper segmentation at the tailbud stage [69]. In the present study, no significant SNPs were found in the regions of any of these genes, and there were no functional SNPs within these genes in the piglets of the present study.

In pigs, anomalies of the tail with a sharp, pronounced angle, known as a “crooked” or “kinky” tail, have been described in previous studies [70,71,72]. Adhesions of the vertebral column, movement disorders, and urogenital anomalies have been observed in association with the occurrence of kinked tails in pigs [54,73,74]. Also in this case, the genetic background and the underlying mechanism of inheritance have not been fully elucidated [70,71,72,73,74,75]. However, a few candidate genes were identified in the region of the SNPs associated with tail length and/or kinks mapped in the present study. These include the serum/glucocorticoid-regulated kinase 1 (SGK1) gene, which has been associated with defective tail morphology in Xenopus [76]. The ribosomal protein S12 (RPS12) has been implicated in reduced growth rates post-partum and kinked tails in mice [77]. The cellular communication network factor 2 (CNN2) gene has also been shown to be associated with notochord development and the expression of anurie, shortened, kinked, and curled tails in mice, xenopus, and zebrafish [78,79,80]. All of these genes showed significant pleiotropic effects affecting a wide range of organ systems and in some cases were associated with embryonic lethality. CCN2 was also associated with growth and developmental regulation [81], carcass composition (meat and fat) [82], and reproduction [83] in pigs. This observation raises the possibility that the SNP on SSC1 identified in the present study may have arisen by selection sweep [84].

Of particular interest are two of three 100% linked SNPs in the protocadherin A1 (PCDHA1) gene, which are associated with two amino acid exchanges. These SNPs were mapped as putative SNPs on SSC2 and were significantly associated with relative tail length and the occurrence of kinks (0/1). It is not clear how cadherins/protocadherins might specifically contribute to the described changes in piglet tails, but the cadherin family has been shown to play important roles in controlling morphogenesis and cell biological properties during various developmental processes in vertebrates [85,86]. Mutations in genes in this family have been associated with various forms of malformations, including those of the brain, ears [86], diaphragm, and lungs [87]. However, further basic research is needed to establish a direct link between PCDHA1 and tail abnormalities in piglets.

The SNP on SSC6 in an intron of the JAK1 gene provides little evidence for a physiological link to shortened or kinked tails in piglets. JAK1 is a key regulator of the immune system, involved in processes ranging from inflammation to immune response. Direct effects on tail shortening or kinking are not apparent. In pigs, the JAK1 region on SSC6 has been associated with litter size and carcass backfat thickness [88,89,90]. However, in both cases, this is likely to be a linkage to another unknown SNP on SSC6 [89]. This is especially true for the coupling of JAK1 with the leptin receptor (LEPR).

The PCDH17 gene, located in the region of the SNP on SSC 11, belongs to the protocadherin gene family and its methylation level has been suggested to be important for tumour progression in several cancers and is shown to be associated with body weight and growth [91,92,93].

The frizzled class receptor 5 (FZD5) gene, which is involved in the hippo signalling pathway, is located in the region around the SNP on SSC 15. This pathway has been implicated in the regulation of tail regeneration in invertebrates and vertebrates [94]. Furthermore, FZD5 was found to be involved in embryonic development in pigs [95,96].

The relatively small number of animals is a limiting factor of the present study and caution is needed when interpreting the results as it reduces the statistical power of the GWAS. This decreases the likelihood of detecting true effects and the precision of the mapping [97]. However, the present study was designed to investigate whether chromosomal regions with larger effects could be identified at all. Finally, the results are based on 348 animals. By selecting all piglets of the larger cohort with kinky tails and at the same time selecting the most extreme piglets in terms of tail length, two questions could be answered in parallel using the same group of animals. Furthermore, by selecting the extremes, the variance in the 348 animals was maintained despite the reduction in the actual genomically sequenced piglets to 140 animals, simply by not sequencing the piglets with medium tail lengths.

Also, the exclusion of candidate genes from other studies and other species is less dependent on the number of animals available as no evidence of functional SNPs was found in the sequence regions of these genes, while a small number of animals would only have affected the significance of the effects and the exact localisation within the candidate genes. GWASs with significantly fewer animals are available in the literature (e.g., [98]). Based on the present results, future studies with larger numbers of animals may succeed in fine-mapping the genomics of tail length and associated kinks in pigs.

## 5. Conclusions

A number of candidate genes have been associated with tail length traits in different animal species, often in association with the occurrence of malformations. Despite small effects on tail length, the SNPs identified by the present study were also strongly associated with the occurrence of tail malformations in the form of kinks. The abovementioned candidate genes, which are often associated with tail length or the occurrence of kinks in other studies and species, were not significant for the effects in the present study. Nevertheless, there was evidence for candidate genes in the region of the identified SNPs with a physiological correlation to corresponding tail alterations in mouse or xenopus. However, further studies are needed to substantiate these associations and to prove causality. The close association between tail length and kinks and the small effect on tail length at the SNP level indicate that the phenotype studied is not suitable for improving animal welfare and health by selecting for shorter tails.

## Figures and Tables

**Table 1:** Phenotypic data from all 348 piglets of the cohort compared to the 140 piglets that were used for GWAS.

Total piglets (n = 348)				
	Mean	SD	Min	Max
Litter size	17.5	3.7	5	24
Sex (0 = male; 1 = female)	0.5			
Parity	2.7	1.8	1	6
Body length (cm)	25.5	2.4	18.0	31.5
Tail length (cm)	9.5	1.1	6.3	12.0
Total length (cm)	35.1	3.2	25.2	42.1
Relative tail length (%)	27.2	1.8	20.8	31.0
Kinks (% piglets with kinks)	12.0			
Kink grade (°)	4.9	18.2	0	180
Piglets for GWAS (n = 140)				
	Mean	SD	Min	Max
Litter size	17.4	3.4	10	24
Sex (0 = male; 1 = female)	0.5			
Parity	2.6	1.7	1	6
Body length (cm)	26.0	2.4	18.0	31.5
Tail length (cm)	9.91	2.1	6.3	12.0
Total length (cm)	35.5	3.1	26.1	42.1
Relative tail length (%)	26.7	2.1	20.8	31.0
Kinks (% piglets with kinks)	30.0	0.5		
Kink grade (°)	12.4	27.3	0	180

**Table 2:** Association between tail length and kinks in the 140 GWAS piglets.

	20% Shortest Tails	60% Piglets Intermediate Tail Length	20% Longest Tails
Total piglets (n = 348)			
Mean relative tail length (%)	24.30	27.30	29.40
Min relative tail length (%)	20.20	25.80	29.80
Max relative tail length (%)	25.80	28.60	31.30
Percentage piglets with kinks (%)	28.00	9.00	5.00
Mean kink grades (°)	13.60	3.40	1.60
SD kink grades (°)	34.20	12.30	6.90
Piglets for GWAS (n = 140)			
Mean relative tail length (%)	23.89	27.06	29.68
Min relative tail length (%)	20.82	26.52	28.99
Max relative tail length (%)	25.00	27.71	31.03
Percentage piglets with kinks (%)	54.00	29.67	7.00
Mean kink grades (°)	25.71	11.54	2.22
SD kink grades (°)	8.69	3.79	1.54

**Table 3:** SNPs significantly associated with relative tail length, prevalence (kinks 01), and severity of tail kinks (kink grades) in GWAS.

SSC	Position	Trait	*p* #	Impact	Region	Gene 5′	Gene 3′	Nucleotide	Triplet	AA
1	30,699,535 ^1^	kink grades	5.31 × 10^−11^	modifier	intergenic variant	LOC106507477	LOC102166614	G/A	-	
2	142,460,474 ^2^	kinks01	8.45 × 10^−6^	moderate	missense_variant	PCDHA1	PCDHA1	C/A	AGC/AGA	Ser/Arg *
2	142,461,085 ^3^	kinks01	6.59 × 10^−6^	moderate	missense_variant	PCDHA1	PCDHA1	A/G	AAG/AGG	Lys/Arg **
2	142,401,326 ^4^	kinks01	8.45 × 10^−6^	low	5′UTR	PCDHA1	PCDHA1	T/C	-	
6	147,382,689 ^5^	relative tail length	3.85 × 10^−12^	modifier	intron variant	JAK1	JAK1	C/A	-	
11	30,485,036 ^6^	kink grades	3.69 × 10^−13^	modifier	intergenic variant	LOC100515964	PCDH17	T/G	-	
15	111,206,597 ^7^	kink grades	7.92 × 10^−12^	modifier	intergenic variant	PLEKHM3	LOC100154892	A/G	-	

#: ≤ 3.53 × 10^−9^: genome wide significant; ≤ 1 × 10^−5^: suggestive significant; *: SIFT deleterious_low_confidence (0.05); **: SIFT tolerated_low_confidence (1); SNP-IDs: ^1^ rs331299164;^2^ rs335834834; ^3^ rs320942126; ^4^ rs81366490; ^5^ rs318560582; ^6^ rs331098176; ^7^ rs327057001.

**Table 4:** Relative tail length, prevalence (kinks 0/1), and severity of tail kinks (kink grades) by SNP genotype.

Genotype	SSC 1: 30,699,535	AA	GA	GG	*p*	R^2^
N		5	27	107		
Rel. tail length (%)	Mean	26.64 a *	27.92 b	27.24 b	0.02455163	25.2
	SE	0.204	0.417	1.048		
	Min	26.234	27.089	25.165		
	Max	27.044	28.742	29.320		
Kinky tails (%)	%	35.8 b	28.6 b	0 a	0.039	6.7
Kink degrees (°)	Mean	17.60 b	8.57 b	0 a	0.002	17.8
	SE	2.552	5.210	13.101		
	Min	12.542	−1.762	−25.983		
	Max	22.666	18.905	25.983		
Genotype	SSC 2: 142,460,474	CC	CA	AA	*p*	R^2^
N		6	45	87		
Rel. tail length (%)	Mean	24.53 a	26.53 b	27.24 b	0.025	30.4
	SE	1.00	0.64	0.60		
	Min	22.52	25.15	25.93		
	Max	26.54	27.90	28.60		
Kinked tails (%)	%	83.3 a	44.4 b	18.4 c	0.000107	7.8
Kink degrees (°)	Mean	58.88 a	26.01 b	12.05 c	0.003	68.9
	SE	12.79	6.64	5.90		
	Min	33.56	12.87	0.37		
	Max	84.20	39.16	23.74		
Genotype	SSC 6: 147,382,689	AA	CA	CC	*p*	R^2^
N		0	14	126		
Rel. tail length (%)	Mean		28.63 a	26.28 b	0.000	31.3
	SE		0.66	0.36		
	Min		27.33	25.58		
	Max		29.93	26.99		
Kinked tails (%)	%		43.10	49.10	n.s.	2.7
Kink degrees (°)	Mean		21.76	23.84	n.s.	9.2
	SE		9.66	5.26		
	Min		2.63	13.44		
	Max		40.87	34.25		
Genotype	SSC 11: 30,485,036	GG	GT	TT	*p*	R^2^
N		121	16	3		
Rel. tail length (%)	Mean	26.96 a	27.04 a	24.28 b	0.06322053	30.4
	SE	0.22	0.47	1.11		
	Min	26.51	26.10	22.08		
	Max	27.40	28.00	26.49		
Kinked tails (%)	%	21 a	47.9 b	100 c	0.00312586	7.8
Kink degrees (°)	Mean	6.36 a	19.38 b	135 c	2.1317 × 10^−24^	68.9
	SE	1.86	3.93	9.25		
	Min	2.68	11.58	116.66		
	Max	10.04	27.17	153.34		
Genotype	SSC 15: 111,206,597	GG	GA	AA	*p*	R^2^
N		2	10	128		
Rel. tail length (%)	Mean	22.44 a	26.01 b	26.87 b	0.015	27.8
	SE	1.49	0.71	0.38		
	Min	19.48	24.61	26.12		
	Max	25.40	27.42	27.62		
Kinked tails (%)	%	100.00	56.00	14.14	0.028	4.6
Kink degrees (°)	Mean	187.5 a	21.21 b	3.76 b	1.04 × 10^−19^	56.6
	SE	14.83	7.04	6.70		
	Min	158.13	7.28	21.59		
	Max	216.88	35.15	13.77		

*: values with different letters are statistically significant (*p* < 0.05).

**Table 5:** Relative tail length, prevalence (kinks 0/1), and severity of tail kinks (kink grades) by number of genotype (0 to 3) associated with tail shortness and tail kinks at the SNPs on SSC1, 2, 11, and 15.

N Short Tail/Kink-Associated Genotypes		0 *	1 *	2 *	3 *	*p*
	N	125	9	2	1	
Rel. tail length	Mean	27.03 a	26.72 a	24.81 b	22.10 b	0.05
	SD	2.12	1.82	0.87		
	Cilow **	26.66	25.32	16.99		
	CIup ***	27.41	28.13	32.62		
Kinks	(%)	28	33	100	100	0.063
Kink grades	Mean	9.36	10	135	180	4.4314 × 10^−25^
	SD	17.22	15	63.64		
	CIlow	6.31	−1.53	−436.78		
	CIup	12.41	21.53	706.78		

*: 0, 1, 2, 3: individuals with no, 1, 2, or 3 genotypes at the SNPs on SSC1, 2, 11, and 15, associated with short or kinky tails, respectively. **: lower 95% confidence interval; ***: upper 95% confidence interval. Values with different letters are statistically significant (*p* < 0.05).

## Data Availability

Data are available from the corresponding author on reasonable request.

## Supplementary Materials

The following supporting information can be downloaded at: https://www.mdpi.com/article/10.3390/vetsci12030198/s1. Supplemental Table S1: Modified config.yaml-file used for the Ovarflow workflow; Supplemental Figure S1: PCA plot with comparison of PC1 (x-axis) and PC2 (y-axis) and indication of the explained variance of each PC. The offspring of the six boars are shown in different colours.

## Abbreviations

The following abbreviations are used in this manuscript:

ACVR2B	Activin A receptor type 2B
ANKRD11	Ankyrin repeat domain containing 11
CI	Confidence interval
CNN2	Cellular communication network factor 2
FZD5	Frizzled class receptor 5
GWAS	Genome wide association study
HES7	HES family bhlh transcription factor 7
HOXB	Homeobox B
LIN28A	LIN-28 homolog A
INDEL	Insertion-deletion polymorphism
JAK1	Janus kinase 1
LEPR	Leptin receptor
OH	Oberer Hardthof teaching and research station of the Justus-Liebig-University Giessen
PAB	Pseudoautosomal boundary
PAR	Pseudoautosomal region
PAX	Paird box
PCA	Principal component analysis
PCV2	Porcine Circovirus Type 2
PCDH17	Protocadherin 17
PCDHA1	Protocadherin A1
PLEKHM3	Pleckstrin homology domain containing M3
QTL	Quantitative trait loci
RPS12	Ribosomal protein S12
SFRP2	Secreted frizzeled related protein 2
SGK1	Serum/glycocorticoid kinase 1
SNP	Single nucleotide polymorphism
SSC	Sus scrofa chromosome
SIFT	Sorting intolerant from tolerant score
VEGFA	Vascular endothelial growth factor A
VSD	Vertebral and spinal dysplasia
WGS	Whole genome sequence
WNT3A	WNT family member 3A
