# Supplementary material for: Genome-Wide Association Study Reveals Single Nucleotide Polymorphisms Associated with Tail Length and Tail Kinks in Piglets
Source: Vet Sci. 2025 Feb 24;12(3):198. doi: 10.3390/vetsci12030198 (PMC11946323; doi:10.3390/vetsci12030198)
Supplement: Supplementary file 1 [file vetsci-12-00198-s001.zip › vetsci-3468646-supplementary.pdf]

veterinary sciences

GWAS Reveals SNPs Associated with Tail Length and Tail Kinks in Piglets

Katharina Gerhards [1], Christiane Egerer [1], Sabrina Becker [1], Hermann Willems [1], Petra Engel [2], Sven König[2] and Gerald Reiner [1,*]

Supplementary Table S1. Modified config.yaml file used for the OVarFlow workflow; the original default settings are shown in brackets.

Config.yaml	
# yaml file listing optionally available configuration	
# no option nor the yaml file itself must be present	
# here the default options of OVarFlow are listed	
**heapSize:**	
SortSam:	**10**
MarkDuplicates:	**4** (2)
HaplotypeCaller:	**3** (2)
GatherIntervals:	**2**
GATKdefault:	**12**
**ParallelGCThreads:**	
SortSam:	**2**
MarkDuplicates:	**2**
HaplotypeCaller:	**2**
GatherVcfs:	**2**
CombineGVCFs:	**2**
GATKdefault:	**4**
**Miscellaneous:**	
BwaThreads:	**6**
BwaGbMemory:	**8** (4)
GatkHCintervals:	**4**
HCnpHMMthreads:	**4**
GATKtmpDir:	**"./GATK_tmp_dir/"**
MaxFileHandles:	**300**
MemoryOverhead:	**1**
**Debugging:**	
CSV:	**False**
YAML:	**False**

 https://doi.org/10.3390/vetsci12030198

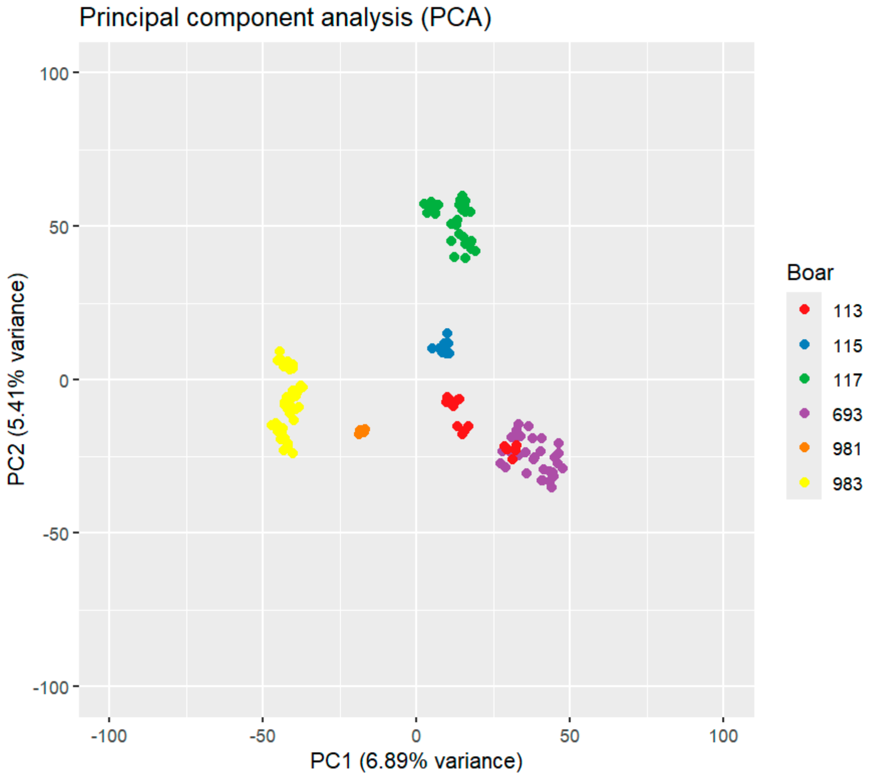

Supplementary Figure S1. Results of principal component analysis. Offspring of the different boars are shown with different symbols. Explained variance was 12.3%. Effects were not included into the GWAS model in favour of the kinship matrix..
